# An eye‐catching atypical illustration of the evaluation and management of AL amyloidosis secondary to myeloma

**DOI:** 10.1002/ccr3.5176

**Published:** 2021-12-09

**Authors:** Shamis Khan, Sarah Premji, Quillan Huang, Gordana Verstovsek, Sita Bushan, Sarvari Venkata Yellapragada

**Affiliations:** ^1^ Internal Medicine Baylor College of Medicine Houston Texas USA; ^2^ Hematology and Oncology Michael E DeBakey VA Medical Center Houston Texas USA; ^3^ Section of Hematology & Oncology Baylor College of Medicine Houston Texas USA; ^4^ Baylor College of Medicine Dan L Duncan Comprehensive Cancer Center Houston Texas USA

**Keywords:** AL amyloidosis, autologous stem cell transplant, myeloma, remission, SLiM‐CRAB criteria

## Abstract

We present a case of a 58‐year‐old male patient who presented to his primary care clinic with complaints of eye swelling and fatigue. Workup ultimately led to a diagnosis of AL amyloidosis secondary to myeloma based on SLiM‐CRAB criteria. We discuss his diagnostic workup, treatment, and subsequent relapse.

## Graphical abstract

In the setting of AL amyloidosis, relapse can occur despite initial complete resolution, and treatment is guided by patient factors, disease factors, and treatment toxicity.

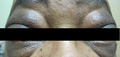

## 1 INTRODUCTION

### 1.1 Background

Amyloidosis collectively refers to a group of diseases that are characterized by an excess of amyloidogenic protein that misfolds, aggregates, and deposits into several tissues causing organ dysfunction.^1^ AL amyloidosis, although the most common form of amyloidosis in developed countries, is still a rare entity, with an annual incidence of roughly 10 patients per million, an estimated prevalence of 12,000 adults in the United States currently, and a mean age of diagnosis of 63 with a greater predisposition for males.^2^, ^3^, ^4^

### 1.2 Pathogenesis

Amyloid is composed of highly ordered protein fibrils, some of which are functional, while others are pathological. While many types of systemic amyloidosis exist, a shared characteristic is that the amyloidogenic protein will be expressed in one or more tissues, circulate in the bloodstream, and deposit in a number of target organs. AL amyloidosis, in particular, is characterized by a plasma cell clone capable of producing a light chain with mutations in the variable region, which leads to improper aggregation and oligomer formation.^1^

### 1.3 Clinical manifestations

AL amyloidosis most commonly affects the heart (75%) and kidney (65%), followed by soft tissues (15%), liver (15%), peripheral/autonomic nervous systems (10%), and GI tract (5%). Thus, clinical manifestations can include signs and symptoms of heart failure, nephrotic syndrome, fatigue, weight loss, peripheral and autonomic neuropathy, hepatomegaly, GI disturbances, and coagulopathy, among other findings. Given these nonspecific signs, almost 40% of cases are diagnosed one year after symptom onset. Given the rapid nature of the disease, roughly 30% of subjects develop irreversible organ damage and die within a year of diagnosis.^5^

### 1.4 Diagnosis

The process of diagnosing AL amyloidosis comprises confirming amyloid deposition, typing deposits, and assessing extent of the monoclonal disease. Generally, the process begins with tissue biopsy and Congo red staining, commonly with an abdominal fat aspirate. If this is negative, it can be followed by a salivary gland biopsy or biopsy of the affected organ after careful evaluation of hemostasis. The gold standard for typing deposits is mass spectrometry, and immune‐electron microscopy or immunohistochemistry in specialized laboratories are reasonable alternatives. Subtyping is crucial as several different proteins are capable of causing systemic amyloidosis, and each warrants a different treatment plan. If a hereditary etiology is suspected, gene sequencing should be performed as well.^2^

The next steps involve assessing burden and extent of clonal and organ disease. Identification of amyloidogenic plasma cell clone requires combination immunofixation of serum and urine with measurement of circulating free light chain. A bone marrow aspirate or biopsy should be performed to evaluate for myeloma. Additionally, skeletal studies should be performed to determine bone involvement. Organ involvement warrants obtaining several biomarkers, an electrocardiogram, and performing a thorough imaging workup.^2^, ^6^

### 1.5 Prognosis

Prognosis is based on recent guidelines which have developed a four‐stage system based on various biomarkers that help illustrate the extent of disease. These include troponin‐T (cTNT) ≥0.025 ng/ml, N‐terminal pro‐B‐type natriuretic peptide (NT‐ProBNP) ≥1,800 pg/ml, and the difference between involved and uninvolved light chain (FLC‐diff) ≥18 mg/dl. Patients are assigned a score of 1 for each of these parameters, creating stages I, II, III, and IV with median overall survival being 94.1, 40.3, 14, and 5.8 months, respectively. This system demonstrates the significance of cardiac involvement in determining survival and guiding management options.^7^

### 1.6 Treatment and response

The premise of treatment in AL amyloidosis revolves around the suppression of synthesis of amyloid precursors prior to the dissemination and deposition of misfolded protein aggregates throughout the body. With recent advances in treatment, 30%–40% of patients are now surviving greater than 10 years.^1^ Treatment is based on the patient's risk and stage of disease; in lower‐risk patients, aggressive therapies are appropriate, whereas in high‐risk patients with low life expectancy, treatment must be tailored toward gentle yet rapidly acting regimens.^2^ The variety of regimens that can be used in both stem cell eligible and non‐eligible patients is listed in Table 1.

**TABLE 1:** NCCN approved regimens in primary treatment of systemic light chain amyloidosis^21^

Preferred Regimens for Systemic AL Light Chain Amyloidosis in Stem Cell Transplant Eligible and Ineligible Candidates
Daratumumab and hyaluronidase–fihj.bortezomib/cyclophosphamide/dexamethasone (preferred)
Bortezomib ± dexamethasone
Bortezomib/cyclophosphamide/dexamethasone
Bortezomib/lenalidomide/dexamethasone
Bortezomib/melphalan/dexamethasone
Melphalan/dexamethasone

From a hematologic standpoint, the goal is to achieve a complete response (CR), defined as negative serum and urine immunofixation and normal serum free light chain (FLC) ratio. Very good partial response (VGPR) is characterized by a difference between involved and uninvolved FLC (dFLC) <40 mg/L and partial response, an unsatisfactory endpoint, would be a decrease in dFLC >50%. Of note, therapy should be continued following the best hematologic response to reduce chances of relapse. With respect to organ response, various parameters including interval changes in biomarkers such as NT‐proBNP, New York Heart Association (NYHA) HF classification score, and estimated glomerular filtration rate (eGFR) are all utilized to illustrate a patient's response to treatment and help guide further management decisions.^2^, ^6^

Following a risk assessment of patients, specific treatment options can considered. In low‐risk patients which comprise roughly 15%–20% of those with AL amyloidosis, autologous stem cell transplant (ASCT) should be considered if patients meet eligibility criteria as listed in Table 2. These refined criteria for patient selection have helped reduced transplant‐related mortality to <5%.^2^ The efficacy of ASCT is impressive, with a hematologic response rate exceeding 70% and approximately 35% of patients achieving CR with a median survival of 8 years, and 55% of these patients surviving at 14 years.^1^ In patients who otherwise meet criteria but have >10% plasma cells in the bone marrow, current guidelines recommend induction chemotherapy with bortezomib and daratumumab‐based regimens prior to ASCT to improve the chances of attaining CR following ASCT.^1^, ^3^ Overall, this sequential approach leads to a CR of 60%.^6^

**TABLE 2:** Eligibility criteria for autologous stem cell transplant^1^

Parameter	Requirements
Cardiac troponin T (cTnT)	Less than 0.06 ng/ml
NT‐proBNP	Less than 5000 ng/L
Age	Less than 65 years
Performance status	0–2
Ejection fraction	Greater than 45%
Systolic blood pressure (standing)	Greater than 90 mm Hg
CO diffusion capacity	Greater than 50%

For intermediate‐risk patients ineligible for ASCT, comprising roughly 70% of the patient population, non‐transplant chemotherapy is the first‐line treatment choice.^6^, ^8^ Melphalan‐based regimens have recently been replaced by novel therapies as the frontline therapy in AL amyloidosis.^6^ Bortezomib is a proteasome inhibitor that causes a rapid decline in serum FLC concentration, as seen in both patients with multiple myeloma and AL amyloidosis. It can be used in patients with severe renal and cardiac involvement; however, its side effect profile includes neurotoxicity, and thus, it should not be used in the setting of peripheral neuropathy. The hematologic response rates of various bortezomib‐based regimens are impressive, with cyclophosphamide, bortezomib, and dexamethasone (CyBorD) reportedly demonstrating an 81.4% response, followed by bortezomib and dexamethasone (71%), bortezomib and melphalan (67%), and bortezomib alone (69%). Other proteasome inhibitors under investigation include ixazomib and carfilzomib.^8^ Furthermore, the recent groundbreaking ANDROMEDA study has introduced daratumumab, a monoclonal antibody against CD38, as a potential addition to CyBorD based on trials demonstrating an improved hematologic response that is statistically significant with an acceptable safety profile.^9^, ^10^

Several other treatment options are available to patients. For example, immunomodulatory drugs, such as thalidomide, lenalidomide, and pomalidomide, have been a recent addition to the treatment pool. Furthermore, several other treatment options such as anthracycline derivatives, tetracycline derivatives, green tea phenols, antisense oligonucleotides, and small interfering RNA have shown promise and are worthy of consideration.^8^, ^11^

In the high‐risk AL amyloidosis patient population (approximately 15%–20%), the disease progression is often characterized by extensive cardiac involvement, and no treatment approach has been shown to reverse this process that portends a poor prognosis. The median survival ranges from three to seven months. Those that are expected to survive at least three months are offered low‐dose chemotherapy combinations to attempt a prolonged survival. Doses are titrated according to patient tolerability; however, patients must be intensively monitored and shared decision‐making involving goals of care discussions is essential.^6^

### 1.7 Relapse

While limited information exists regarding relapse, various retrospective studies have been published stating that relapse can occur, even in patients who initially had a complete response. Laboratory findings associated with relapse include baseline involved/uninvolved free light chain ratio (IUR) >6 and difference between involved and uninvolved FLC (dFLC) >60 mg/L.^12^ Fortunately, relapsed patients have a relatively good prognosis as compared to those refractory to treatment.^6^ However, patients with organ involvement in the setting of relapse have significantly decreased odds of survival. Thus, monitoring biomarkers and evaluating for organ involvement are the key determinants in initiating additional treatment.^12^ Generally, the therapy which the patient initially responded to should be repeated. If patients are not responsive, bortezomib and daratumumab‐based regimens (if not used as initial therapy) or immunomodulatory agents should be used as second‐line therapy if there are no contraindications. Additionally, the abovementioned proteasome inhibitor, carfilzomib, has shown promise in the relapsed setting, as have ixazomib and the alkylating agent, bendamustine.^2^, ^6^ A list of regimens used in relapsed cases can be found in Table 3.

**TABLE 3:** Common Treatment options in relapsed/refractory cases of systemic light chain AL amyloidosis^21^

Treatment Regimens for Relapsed Systemic AL Light Chain Amyloidosis
High‐dose melphalan with hematopoietic cell transplant
Bortezomib ± dexamethasone
Bortezomib/melphalan/dexamethasone
Daratumumab
Ixazomib ± dexamethasone
Ixazomib/lenalidomide/dexamethasone
Lenalidomide/cyclophosphamide/dexamethasone
Lenalidomide/dexamethasone
Melphalan/dexamethasone
Pomalidomide/dexamethasone
Bendamustine/dexamethasone (useful in special circumstances)
Carfilzomib (for non‐cardiac amyloidosis) ± dexamethasone
Venetoclax (For t(11;14) translocation) ± dexamethasone

## 2 CASE

**FIGURE 1:**
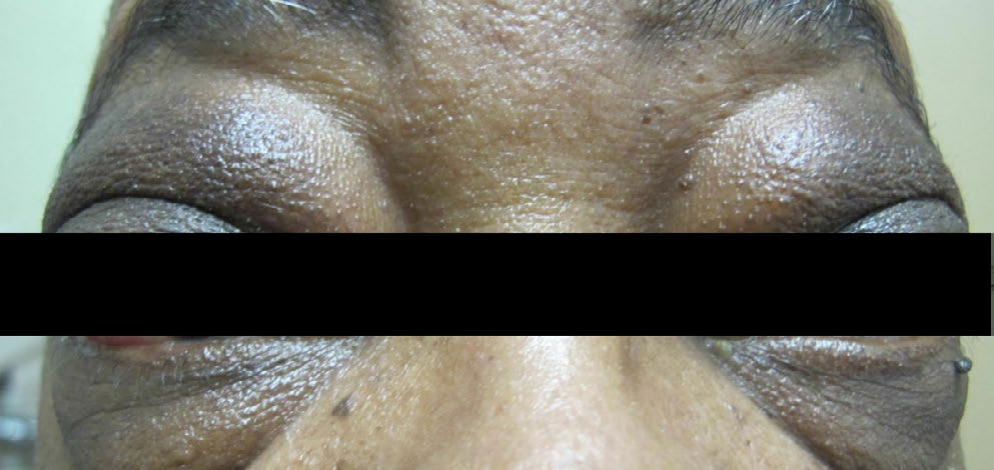
Photograph taken on initial presentation highlighting periorbital fullness

**FIGURE 2:**
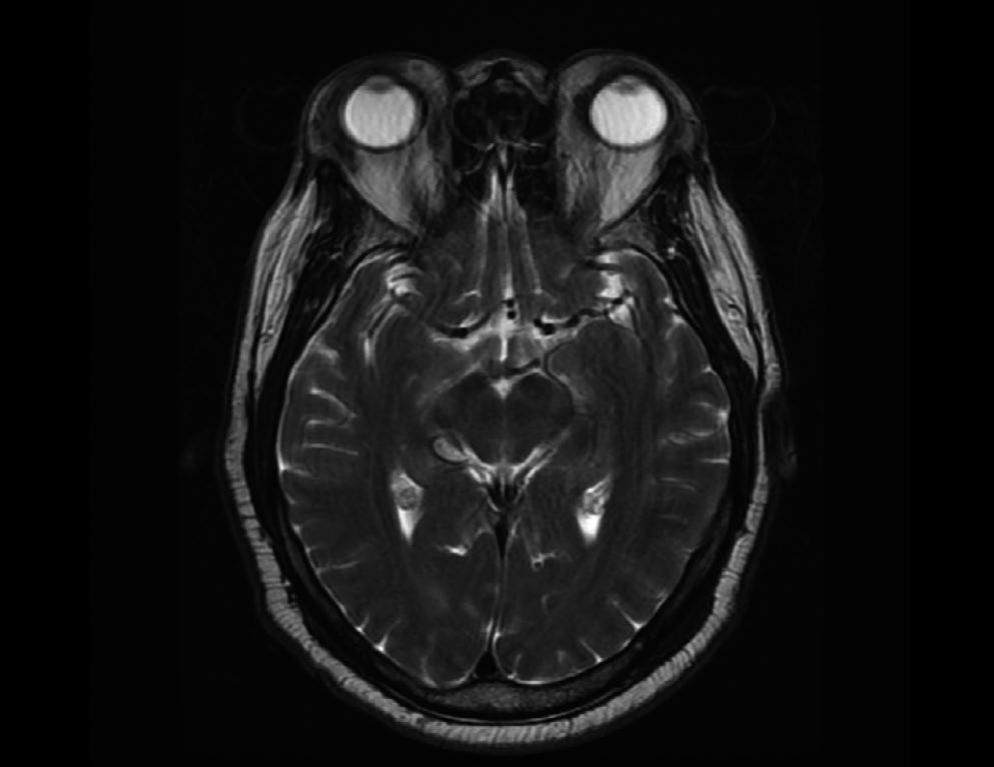
MRI brain/orbit demonstrating bilateral proptosis

**FIGURE 3:**
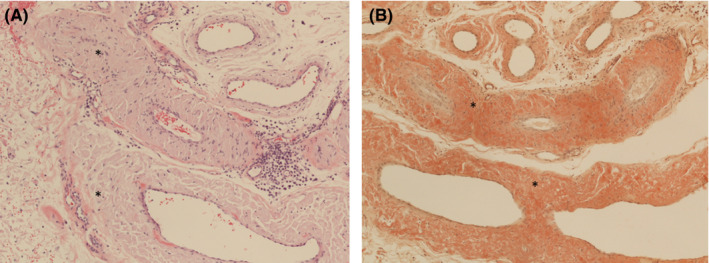
(A) Vessels in periorbital soft tissue show thickening of the wall (star) due to deposits of pink amorphous material within the wall. (B) Congo red special stain confirms that the amorphous material is amyloid. (100×; A Hematoxylin and eosin; B Congo red)

**FIGURE 4:**
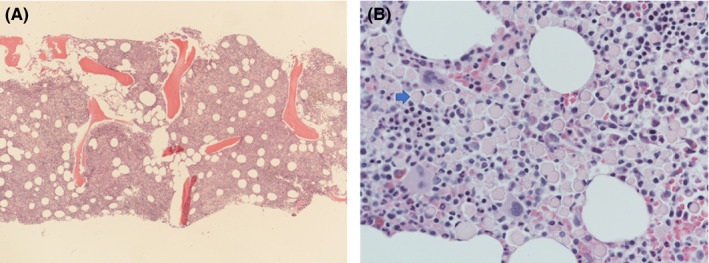
Bone marrow biopsy showing atypical plasma cells with giant intracytoplasmic Russell bodies with nucleus displaced to the side. (4A 40×; 4B 400×; Hematoxylin and eosin)

We present a case of a 58‐year‐old male patient with no past medical history who presented to his primary care clinic with complaints of eye swelling (R > L) and fatigue in June 2015 (Figure 1). Due to worsening swelling, he began to have difficulty with vision due to limited range of motion of his extraocular movements. A CT head demonstrated periorbital fullness for which he was referred to ophthalmology who performed an MRI brain/orbit, which revealed infiltrative disease affecting the eyes bilaterally (Figure 2). Using Congo Red staining, a biopsy was positive for amyloid deposition in the soft tissues and vessels of the eyes (Figure 3). Further workup revealed a lambda light chain of 1345, kappa‐free light chain of 9, no monoclonal spike, and normal cardiac and renal function. A bone marrow biopsy (Figure 4) revealed abnormal plasma cells (5%), and a lower lip biopsy showed microfocal amyloid deposition in the vessel walls, subtyped as AL lambda‐type amyloid.

Our patient diagnosed with Stage I (given lack of cardiac/renal involvement with BNP 60–70s, troponin I < 0.03, and creatinine 0.7–0.9) AL light chain amyloidosis and promptly began treatment with bortezomib and dexamethasone. Due to lack of cardiac or renal involvement, the patient was considered a strong candidate for ASCT. He completed 9 cycles of induction chemotherapy with very good partial response with repeat kappa‐free light chain of 17.39 and lambda‐free light chain of 29.55. Thereafter, he underwent an ASCT in 2016 and a bone marrow biopsy three months afterward showing normal marrow with no amyloid involvement, signifying complete remission. His vision had improved although a CT orbit with contrast continued to show stable infiltrative changes.

**FIGURE 5:**
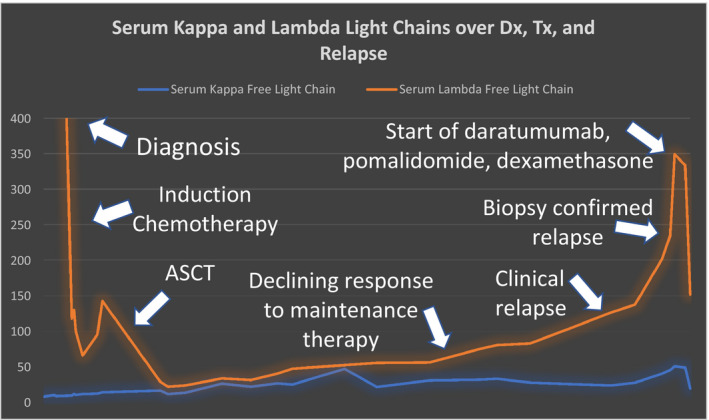
Line graph demonstrating the trajectories of both serum lambda‐free light chain (orange) and serum kappa‐free light chain (blue) from diagnosis to the most recent initiation of therapy for relapse

He was then placed on lenalidomide maintenance therapy, but unfortunately, he was found to have relapsed roughly 4 years later in 2020 when he began noticing eye swelling, ankle swelling, and upper extremity paresthesias. Blood work revealed a kappa‐free light chain of 51.4 and a lambda‐free light chain of 349. A subsequent bone marrow biopsy showed abnormal plasma cells (2% in a limited sample) consistent with a diagnosis of relapsed AL amyloidosis. After relapse, lenalidomide was stopped, pomalidomide was started, and following fluorescence in‐situ hybridization (FISH) studies which revealed an 11;14 translocation (t(11;14)), he was placed on a combination of daratumumab, pomalidomide, and dexamethasone. At his most recent follow‐up in 2021, he noticed improvement in his symptoms and his laboratories showed kappa‐free light chain of 20.4 and a lambda‐free light chain of 151.8. Figure 5 highlights the trends in light chain values throughout the patient's clinical course.

## 3 DISCUSSION

Our patient's chief complaint was simply worsening fatigue and asymmetric eye swelling; however, it ultimately led to a diagnosis of AL amyloidosis. In contrast to secondary (AA) amyloidosis, AL amyloidosis can lead to the deposition of protein aggregates in a variety of ocular tissues such as the conjunctiva, eyelids, extraocular muscles, and even the temporal artery, although this is very rare.^13^ Thus, clinicians must keep a broad differential when patients present atypically, and primary care physicians and specialists must collaborate to most effectively diagnose and treat such conditions. The collaboration of care in this case was instrumental to prolonging the survival of a complicated patient.

From his diagnostic workup, one of the notable findings was an initial free light chain ratio >100. From the most recent guidelines and the newly instituted SLiM‐CRAB criteria, this single‐handedly classifies as a myeloma‐defining event. In this new risk stratification tool used to identify patients at risk of eventually developing multiple myeloma, “S” stands for ≥60% bone marrow plasma cells, “Li” stands for light chain ratio (involved over uninvolved >100), and “M” stands for focal bone marrow deposits >0.5 cm found on MRI. The “CRAB” refers to the commonly known mnemonic associated with multiple myeloma of calcium elevation, renal abnormalities, anemia, and bone disease. Any one of the SLiM findings now constitutes a diagnosis of multiple myeloma. Thus, while the presumptive diagnosis in our patient is AL amyloidosis, it would technically be classified as myeloma‐associated amyloidosis.^14^

Beyond his diagnosis, another unique aspect of our patient's case was his clinical and treatment course. Although his plasma cell involvement was only measured to be 5%, he was treated in accordance with guidelines at the time and started on a bortezomib‐based regimen. He demonstrated VGPR, was an ideal candidate for ASCT given that he did not have significant cardiac or renal involvement and his biomarkers were otherwise not significantly elevated, and showed CR following his transplant. He was started on lenalidomide maintenance therapy, which, in contrast to multiple myeloma in which post‐ASCT maintenance therapy is the standard of care, has not been completely explored in the setting of AL amyloidosis. In the setting of AL amyloidosis, varying evidence exists, but some studies have shown that there is no significant difference between patients that do and do not receive maintenance therapy as measured by FISH bone marrow plasma cell burden (BMPC), progression‐free survival (PFS), and overall survival (OS).^15^ Nonetheless, he did relapse in just a 4‐year time span following his ASCT.

With respect to his treatment course following relapse, often times, treatment for relapse is guided by patient factors, disease factors, and treatment toxicity, and it is paramount to keep these principles in mind when managing relapsed cases. Pomalidomide has shown incredible activity in setting of relapsed multiple myeloma, and it has shown promise in the setting of AL amyloidosis as well.^16^ Furthermore, the agent that was most recently initiated, daratumumab, has proven itself to be a very safe and effective treatment in the setting of relapsed AL amyloidosis, particularly in patients that have previously been treated with bortezomib and lenalidomide‐based regimens.^17^ Lastly, his t(11;14) warrants interest in venetoclax, an oral selective B‐cell lymphoma 2 (BCL‐2) inhibitor that has shown promise in the setting of multiple myeloma. A recent retrospective cohort study assessed 43 patients with relapsed/refractor AL amyloidosis in various centers across the United States and Europe and found venetoclax to promote a better hematologic response in patients with the t(11;14) as opposed to those that did not have it (81% vs. 40%).^18^ Thus, while the reality of relapse is difficult to accept, there is certainly ongoing development geared toward improving treatment options and overall life expectancy.

## 4 CONCLUSION

Our case illustrates an excellent collaborative effort by various medical specialties to identify and treat an atypical presentation of a very rare entity. Unfortunately, our patient did suffer from relapsed AL amyloidosis despite undergoing ASCT. In the relapsed setting, there must be awareness about how a patient previously tolerated therapy and their initial response to it. But often, a new therapy is used (after standard treatment such as a proteosome inhibitor) and some options used in the setting of relapse include daratumumab or IMIDs ^19^, ^20^, ^22^. In envisioning future therapies, aside from chemotherapy or immunotherapy, molecules that inhibit amyloidogenesis, and even doxycycline have been studied in combination with standard treatment and have shown in some small retrospective trials to improve survival in specific cases. Relapse of AL amyloidosis is certainly a topic that requires further investigation, but with continuously updating diagnostic guidelines, prognostic determinants, and novel treatments underway, the future of AL amyloidosis is one we can look at with optimism.

## CONFLICT OF INTEREST

I confirm that none of the authors have any conflicts of interest in the submission of this manuscript.

## AUTHOR CONTRIBUTIONS

All authors listed were involved in the care and/or research of the patient discussed in this case report with Dr. Sarvari Venkata Yellapragada overseeing and guiding our efforts.

## CONSENT

I confirm that the patient consent has been signed and collected in accordance with the journal's patient consent policy. I will retain the consent form and will provide it if requested. As required, patient anonymity has been preserved throughout this manuscript.

## Data Availability

Data sharing not applicable to this article as no datasets were generated or analysed during the current study.
